# Development of GMP‐1 a molecular chaperone network modulator protecting mitochondrial function and its assessment in fly and mice models of Alzheimer's disease

**DOI:** 10.1111/jcmm.13624

**Published:** 2018-04-27

**Authors:** Pavel F. Pavlov, Birgit Hutter‐Paier, Daniel Havas, Manfred Windisch, Bengt Winblad

**Affiliations:** ^1^ Division of Neurogeriatrics Department of Neuroscience Care and Society Karolinska Institutet Huddinge Sweden; ^2^ GreatMatterPharma AB Solna Sweden; ^3^ QPS Austria GmbH Grambach Austria; ^4^ NeuroScios GmbH Radegund/Graz Austria

**Keywords:** Alzheimer's disease, dicarboxylate clamp, mitochondria, molecular chaperones, protein import, tetratricopeptide repeat proteins

## Abstract

Mitochondrial dysfunction is an early feature of Alzheimer's disease (AD) and may play an important role in the pathogenesis of disease. It has been shown that amyloid beta peptide (Aβ) and amyloid precursor protein (APP) interact with mitochondria contributing to the mitochondrial dysfunction in AD. Prevention of abnormal protein targeting to mitochondria can protect normal mitochondrial function, increase neuronal survival and at the end, ameliorate symptoms of AD and other neurodegenerative disorders. First steps of mitochondrial protein import are coordinated by molecular chaperones Hsp70 and Hsp90 that bind to the newly synthesized mitochondria‐destined proteins and deliver them to the protein import receptors on the surface of organelle. Here, we have described the development of a novel compound named GMP‐1 that disrupts interactions between Hsp70/Hsp90 molecular chaperones and protein import receptor Tom70. GMP‐1 treatment of SH‐SY5Y cells results in decrease in mitochondria‐associated APP and protects SH‐SY5Y cells from toxic effect of Aβ_1‐42_ exposure. Experiments in drosophila and mice models of AD demonstrated neuroprotective effect of GMP‐1 treatment, improvement in memory and behaviour tests as well as restoration of mitochondrial function.

## 1 INTRODUCTION

Accumulation of protein or peptide aggregates in the affected brain areas is a molecular hallmark of several neurodegenerative disorders including AD.^1^ Increasing body of evidence suggests that protein misfolding and aggregation are prominent events in the initiation of the pathogenic cascades that occur in degenerated neurons and mitochondria are heavily engaged in this process.^1^ Misfolded proteins can exert a multiplicity of noxious effects on mitochondria. They can directly affect mitochondria from the surface interacting with components of mitochondrial protein import machinery;^2^, ^3^, ^4^, ^5^ in other cases, they interact with components of respiratory chain complexes, mitochondria permeability transition pore or redox enzymes.^6^, ^7^, ^8^, ^9^, ^10^ In AD, both APP and Aβ directly associate with mitochondria causing their dysfunction. APP possess a cryptic mitochondrial targeting signal followed by typical endoplasmic reticulum signal peptide whereas more C‐terminally located domain enriched with acidic amino acids acts as translocation arrest sequence.^11^ Upon mitochondrial entry, APP forms stable translocation intermediate complexes with mitochondrial translocase of the outer membrane (TOM) as well as links together translocases the outer and the inner membranes.^4^, ^11^ Complexes between mitochondrial protein translocases and APP were particularly abundant in the affected areas of the brains of patients with AD suggesting connection between mitochondrial APP accumulation and AD pathology in the brain.^4^ APP accumulated in the mitochondrial import channels can be degraded by several mitochondrial proteases including HtrA2/Omi and gamma secretase.^5^, ^12^ At present, factors contributing to mitochondrial APP accumulation under AD conditions are not known. Genetic variations in components of mitochondrial protein translocase can result in lower efficiency of mitochondrial protein import and APP accumulation in the import channels.^13^ AD‐associated mutations in the mitochondrial protein degradation system^14^ can shift the APP uptake/degradation balance towards mitochondrial APP accumulation. Different environmental factors can also contribute to the mitochondrial APP accumulation. For example, high intracellular cholesterol level, a risk factor for development of Alzheimer's disease, has been shown to affect APP secretion, post‐translational modification and intracellular trafficking.^15^ Although the molecular determinants for Aβ mitochondrial import are not known, mitochondrial protein translocase also participates in the import of Aβ peptide^16^ suggesting that reduction in APP and Aβ mis‐targeting can promote neuronal survival under AD conditions.

Nuclear‐encoded proteins destined to mitochondria enter the organelle via the TOM complex consisting of protein‐conducting channel formed by Tom40, two primary receptors, Tom20 and Tom70, the central receptor Tom22 and several adaptor subunits of small molecular mass.^17^ The majority of mitochondrial precursor proteins enter the organelle via Tom20 receptor whereas hydrophobic mitochondria‐destined proteins with internal targeting signals preferentially follow Tom70 receptor pathway.^17^ Cytosolic molecular chaperones Hsp70 and Hsp90 as well as other specific factors in cytosol participate in targeting of newly synthesized proteins to the Tom70 receptor.^18^, ^19^ The interaction is mediated by the short C‐terminal acidic peptide—EEVD identical in Hsp70 and Hsp90 which binds to the tetratricopeptide repeat motif (TPR) of Tom70 receptor by dicarboxylate clamp mechanism.^19^, ^20^ TPR domain is generally involved in protein‐protein interactions and from 736 TPR motif‐containing proteins annotated in the human UniProt database (http://www.uniprot.org) ~20 different proteins interact with Hsp70 and Hsp90 via dicarboxylate clamp mechanism.^21^, ^22^ APP was previously shown to interact with both Hsp70 and Hsp90;^23^ moreover, we have observed that anti‐Tom70 antibody treatment of isolated mitochondria inhibited Aβ uptake by 60%;^16^ therefore, inhibition of Hsp70/Hsp90 interactions with Tom70 can potentially decrease APP and Aβ mitochondrial load in AD conditions. Available crystal structures of several TPR domain‐containing proteins including Tom70 with Hsp70/Hsp90‐derived peptides revealed relatively small interaction area within the binding site providing opportunity for development of competitive inhibitors of Hsp70/Hsp90‐ Tom70 interactions. As mentioned above, only small fraction of mitochondria‐destined proteins follows Tom70 import pathway suggesting that such inhibitors would not generally impair protein import into mitochondria.

## 2 MATERIALS AND METHODS

### 2.1 In silico docking of virtual compound libraries

In silico docking was performed with DOCK Blaster, a free virtual compound library screening server. PDB structures of TPR proteins cocrystallized with Hsp70/Hsp90 C‐terminal acidic peptides were used for structure‐based ligand discovery. Following structures have been used: Tom70 PDB ID: 2GW1, CHIP: 3Q49, PP5: 2BUG, FKBP52: 1QZ2, AIP: 4AIF. Best‐scoring compounds were purchased from ChemBridge Inc. San Diego, CA, USA, and Vitas‐M Laboratory Ltd., Apeldoom, the Netherlands.

### 2.2 Chemicals

All common chemicals were purchased from Sigma (St. Louis, MO, USA) unless stated otherwise.

### 2.3 Peptides and peptide coupling to HRP

Amyloid beta peptide 1‐42 (Aβ_1‐42_) (H‐1368) was obtained from Bachem Bubendorf, Switzerland. Peptide with sequence NH_2_‐HHHHHHDDTSRMEEVD‐COOH containing 6 × His tag and 10 amino acids corresponding to the C‐terminus of human Hsp90 alpha (C90) was synthesized at GL Biochem, Shanghai, China. Peptide coupling to horse‐radish peroxidase (HRP) was performed according to the kit instructions (Lightning‐link^®^ HRP labeling kit cat. 701‐004; Innova Biosciences). C90 peptide was dissolved in HEPES‐KOH buffer pH 7.2 at concentration 1 mg/mL and incubated with equimolar amounts of activated HRP for 5 minutes at room temperature. Reaction was quenched with 0.1 mol/L Tris‐HCl, pH 7.5 for 10 minutes, and the products were passed through PD‐10 columns (GE Healthcare, Uppsala, Sweden).

### 2.4 Cloning and purification of human TPR proteins Tom70, Tom34, FKBP51 and PP5

Full‐length cDNA clones for respective proteins were obtained from IMAGE consortium (SourceBioScience, UK) and PCR amplified with forward and reverse primers containing overhangs with appropriate restriction sites and cloned into pGEX6 vectors (GE Healthcare, Uppsala, Sweden). Full‐length Tom34, FKBP51 and PP5 proteins as well as cytoplasm‐exposed domain of Tom70, amino acids 65‐608, were amplified. Inserts were sequence verified, and the plasmids were transformed into BL21 *Escherichia coli* strain. Gluthatione‐S‐transferase (Gst) fusion proteins were purified from 1L overnight culture after 2 hours of induction with 1 mmol/L IPTG. Cells were pelleted, resuspended in 1× PBS and sonicated 3 × 20 seconds on ice. EDTA and protease inhibitor cocktail were added to prevent proteolysis. The suspension was centrifuged for 30 minutes 50 000 × *g* to remove cell debris, and supernatant was loaded onto 1 mL Gst‐trap 4B column (GE Healthcare Uppsala, Sweden). After column washing with 30 mL PBS Gst fusion, proteins were eluted with 2.5 mL 10 mmol/L gluthatione in PBS. Eluate was passed through PD‐10 column (GE Healthcare, Uppsala, Sweden) to remove free gluthatione. To prepare Gst‐tag‐free proteins, we performed overnight cleavage at 4°C with PreScission protease (GE Healthcare, Uppsala, Sweden) followed by passage through Gst‐trap column to remove free Gst protein. Protein‐containing fractions were collected, checked with SDS‐PAGE and kept at −20°C.

### 2.5 Dot‐blot experiments using C90‐HRP

Dot‐blot experiments were performed with Gst‐tag‐free proteins applied to the nitrocellulose membrane. After blocking of membrane with 3% BSA in TBS‐T buffer for 1 hour and subsequent wash with TBS‐T, C90‐HRP (1 × 500 times dilution) was applied and incubated for 1 hour. After subsequent washing (3 times 10 minutes, TBS‐T), membrane was dried and ECL signals were quantified using a digital Fujifilm LAS3000 imager and LAS3000 software. In competition, experiments increasing concentrations of test molecules were coincubated with C90‐HRP conjugates. Experiments were performed in triplicates; data are presented as mean ± SEM. *P * < .05 was considered to be statistically significant.

### 2.6 Antibodies

The following antibodies were used anti‐APP 22c11 (MAB348) was from Millipore, Temecula, CA, USA, and Tom40 (Sc‐11414) and Tom70 (sc‐390545) were from Santa Cruz Biotechnology, CA, USA, anti‐APP (6E10) and Aβ_1‐42_ (SIG‐39142) from Covance, USA; CD11b (ab75476) and GFAP (ab7260) were from Abcam Cambridge, MA, USA.

### 2.7 Cell culture, cell fractionation and coimmunoprecipitation experiments

SH‐SY5Y human neuroblastoma cells were cultured in DMEM supplemented with 10% FBS in 5% CO_2_, 95% air at 37°C. Cell fractionation was performed as described in Ref. 5. Immunoprecipitation was performed from SH‐SY5Y cells (0.2 mg of protein) treated with DMSO or 50 μmol/L of GMP‐1 for 12 hours. Cells were scraped from the surface in the presence of TBS with 0.2% Triton X‐100 and protease inhibitor cocktail Roche Applied Science, Indianapolis, IN, USA. After centrifugation at 20 000 × *g*, 4°C, supernatant was incubated with 0.05 mg/mL of anti‐Tom40 antibodies for 3 hours 4°C followed by addition of protein A sepharose (GE Healthcare, Uppsala, Sweden) and incubation for 1 hour at 4°C. After incubation, sepharose was pelleted by brief centrifugation and washed three times with TBS buffer. To elute proteins, SDS‐loading buffer was applied for the beads and boiled for 5 minutes. Proteins were resolved by SDS‐PAGE, transferred to the nitrocellulose membrane and stained with respective antibodies.

### 2.8 SDS‐PAGE and Western blot analysis

Protein (25 μg) was mixed with 2× SDS sample buffer, boiled for 5 minutes and loaded onto 4%‐12% Bis‐Tris precast gels (ThermoScientific, Rockford IL, USA). The samples were electrophoresed and transferred to the nitrocellulose membrane (Whatman, Maidstone, UK), and proteins of interest were detected with specific antibodies using SuperSignal West Pico enhanced chemiluminescence system (ThermoScientific, Rockford IL, USA). Western blot signals were analysed and quantified using a digital Fujifilm LAS3000 imager and LAS3000 software.

### 2.9 MTT cell viability assay

Cellular toxicity was assessed with MTT Cell Proliferation Kit I, Roche Applied Science, Indianapolis, IN, USA according the manufacturer's protocol. Indicated amounts of Aβ_1‐42_ and GMP‐1 were added directly to the culture media and incubated for 24 hours.

### 2.10 Cytochrome oxidase activity assay

Cytochrome *c* (2.7 mg/mL) was reduced by incubation with DTT, and excess of reducing agent was removed by gel filtration on PD‐10 column (GE Healthcare, Uppsala, Sweden). Cytochrome oxidase activity was measured by decrease in absorbance of ferrocytochrome *c* at 550 nm. Mouse brain mitochondria were isolated according to Ref. 4. Isolated mitochondria from non‐Tg mice, vehicle‐treated 5xFAD mice and 5xFAD mice treated with GMP‐1 were diluted in the buffer containing 10 mmol/L Tris‐HCl, pH 7.0, 0.5 mol/L sucrose, 0,05% Triton X‐100 to concentration 0.5 mg/mL. After addition of ferrocytochrome *c*, latency in 550 nm absorbance was immediately measured. Each group included six mice, and experiments were performed in triplicate. Data are presented as mean ± SEM. *P* < .05 was considered to be statistically significant.

### 2.11 Transgenic drosophila experiments

The fly lines containing single and double copies of a signal‐peptide‐Aβ_42_ transgene were generated as described in Ref. 24. The fly line expressing in the neurons dimer Aβ_42_ peptide connected via a linker of 12 amino acids (T‐Aβ_42_) was generated as described in Ref. 25. Flies were maintained on the standard food containing 1% Agar, 8% Brewer's yeast, 8% fructose, 5% potato dry powder, 0.05% Nipagin, 0.1% ascorbate in humidified atmosphere at 25°C. Indicated amounts of compounds were added directly to the food during solidification. The fly mobility assay represents the percentage of flies that able to cross the line at 8 cm from the bottom of test tube in 10 seconds. Six independent measurements for each group were performed. Data are presented as mean ± SEM. *P *< .05 was considered to be statistically significant. The survival assay in flies expressing tandem Aβ_42_ in the neurons was calculated as percentage of adult flies carrying the transgene, plain wings phenotype, to the total number of hatched flies with plain + curly wings phenotype. Six independent measurements for each group were performed. Data are presented as mean ± SEM. *P *< .05 was considered to be statistically significant.

### 2.12 In vivo mice experiments

Experiments with control and 5xFAD, APP/presenilin‐1 transgenic mice^26^ were performed at QPS CRO facility in Graz, Austria. For the open field test, a plexiglas box (48 × 48 cm; TSE‐System^®^) was used. The infrared photo beams were placed in a 1.4‐cm distance around the box. Each test session lasted for 5 minutes to check the mouse's behaviour in the new surroundings. Testing was performed under standard room lighting conditions during the light phase of the circadian cycle. The contextual fear conditioning test was conducted in an automated box provided by TSE‐Systems, Germany. Mice were trained and tested on 2 consecutive days. On the training day, mice received a foot shock (0.5 mA, 2 seconds) 5 seconds after being placed into the conditioning chamber. Thirty seconds afterwards, they were returned to their home cage again. Twenty‐four hours after training, mice were tested by being returned to the conditioning chamber for 5 minutes without any shock, and freezing behaviour was recorded by the automated system and evaluated separately every minute. Freezing was defined as lack of movement except that required for respiration and is expressed as freezing time in percent of the testing time. At the end of the study, animals were killed, and CSF, blood (plasma) and brains were collected. Mice were anaesthetized by standard inhalation anaesthesia (Isoba^®^, Essex). Following blood‐sampling mice were transcardially perfused with physiological (0.9%) saline, then brains were removed. Histological examination was performed with sagittal cryosections (10‐μm thickness) which have been prepared from fixed frozen hemibrains. The right hemisphere of each mouse was systematically and uniformly sectioned at 12 mediolateral levels (collecting 10 sections per level and discarding the next 20 sections) on a Leica CM 3050S cryotome. Collection of sections started with a random section at approximately 0.2 mm lateral from midline and extended to approximately 3.6 mm lateral (based on the Mouse Brain Atlas). Sections were stored at −20°C until used for histological staining. To analyse different features of histopathology, the following targets were chosen for immunofluorescent labelling and quantitative analysis: astroglia (GFAP), activated microglia (CD11b), β‐amyloid (6E10). Heat antigen retrieval in citrate‐based buffer was used. All measurements except region size are threshold based; thus, objects above certain intensity and above a certain size are automatically detected by ImageProPlus software (v6.2). The measurements are carried out within an area of interest (AOI), which is manually delineated for each slice and each brain region. Values of five slices per animal deriving from five different mediosagittal levels were averaged to an individual mean; group values were calculated using the individual means. Data were tested for normality using a Kolmogorov‐Smirnov test; differences between groups were calculated by one‐way ANOVA followed by a Newman‐Keuls post‐hoc test, the alpha‐error set to 0.05.

## 3 RESULTS

### 3.1 Identification of 2‐(methoxymethyl)pyrimido[1,2‐a]benzimidazol‐4‐ol (GMP‐1) as competitive inhibitor of Hsp90 molecular chaperone interaction with Tom70 TPR co‐chaperone

**Figure 1:**
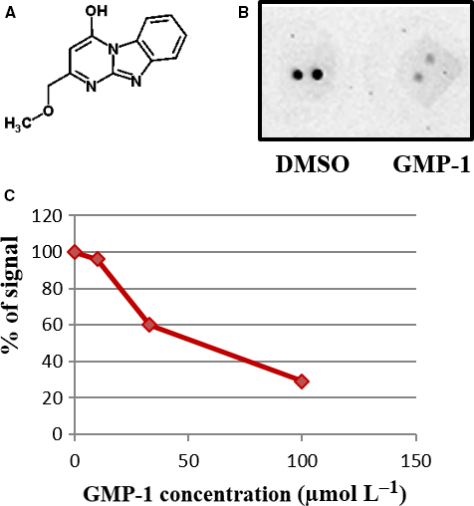
Identification of 2‐(methoxymethyl)pyrimido[1,2‐a]benzimidazol‐4‐ol, GMP‐1 as competitive inhibitor of Tom70‐Hsp90 interactions. A, Chemical structure of GMP‐1. B, Dot‐blot assay of interactions between human Tom70 protein and Hsp90 peptide conjugated to HRP (C90‐HRP) in the presence of DMSO and 100 μmol/L of GMP‐1. C, Dose‐dependent inhibition of Tom70‐ C90‐HRP interactions by GMP‐1. IC_50_ was calculated as a mean value of 3 independent experiments

Protein interaction between molecular chaperones Hsp70/Hsp90 and mitochondrial import receptor Tom70 is mediated by its TPR domain that binds acidic C‐termini of molecular chaperones through dicarboxylate clamp mechanism. We used crystal structures of several TPR proteins including yeast Tom71 cocrystallized with Hsp70/Hsp90 C‐terminal peptides to screen purchasable compound libraries using DOCK Blaster, a free virtual compound library screening server (see Materials and Methods section). For the structure‐based ligand search, we have selected area of peptide binding on the TPR domain. Hundred best‐scoring compounds were purchased from chemical vendors and tested for inhibition of Tom70 interaction with Hsp90 using dot‐blot assay. Tom70 was blotted on nitrocellulose membrane following incubation with Hsp90‐derived peptide (C90) coupled to HRP. One of the compounds, 2‐methylpyrimido[1,2‐a]benzimidazol‐4(1H)‐one, abbreviated as GMP‐1, has inhibitory effect on C90‐HRP interaction with Tom70 in dot‐blot assay in dose‐dependent manner (Figure 1A‐C). Calculated GMP‐1 IC_50_ for Tom70 interaction with C90‐HRP was 45 μmol/L. GMP‐1 was also found among best‐scoring hits for other TPR motif protein, PP5; however, IC_50_ for PP5‐ C90‐HRP interaction was >100 μmol/L. GMP‐1 inhibits C90‐HRP interactions with two other TPR motif proteins, FKBP51 and Tom34, in dot‐blot assay with IC_50 _150 and 180 μmol/L, respectively, indicating relative specificity of GMP‐1 towards Tom70 protein. We have also purchased several structural analogues of GMP‐1 and tested their ability to inhibit Tom70‐ C90‐HRP interactions; however, IC_50 _for these molecules was in the range of 60‐150 μmol/L (data not shown).

### 3.2 Effect of GMP‐1 treatment on APP association with mitochondria in SH‐SY5Y neuroblastoma cell line

**Figure 2:**
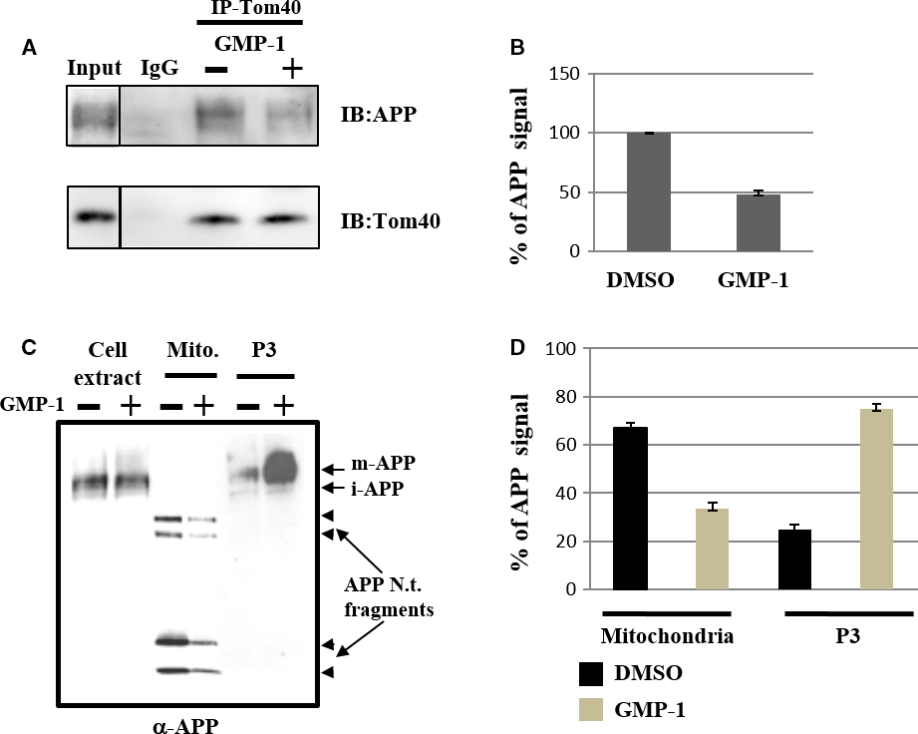
Effect of GMP‐1 treatment on APP association with mitochondria in SH‐SY5Y neuroblastoma cells. A‐B, Coimmunoprecipitation of APP with anti‐Tom40 antibodies. SH‐SY5Y cells were treated with DMSO or 50 μmol/L of GMP‐1 for 16 h. A, Western blot results with anti‐APP (22c11) and anti‐Tom40 antibodies. B, Signal intensity calculated as ratio of APP/Tom40 antibody staining. DMSO‐treated sample was set as 100%. Experiments were performed in triplicates. C‐D, APP association with mitochondria and light membrane P3 fractions isolated from SH‐SY5Y cells treated with DMSO or 100 μmol/L of GMP‐1 for 16 h. C, Western blot results using anti‐APP (22c11) antibody. D, Signal intensity calculated as percentage of staining in mitochondrial or P3 samples to total sample staining. Three independent experiments were performed

Treatment of SH‐SY5Y cells with GMP‐1 up to 100 μmol/L for 48 hours did not result in decrease in cell viability; in contrast, GMP‐1 protects SH‐SY5Y cells from toxic effect of Aβ_1‐42_ exposure (data not shown). Next, we have investigated effect of GMP‐1 treatment on association of APP with mitochondria. As it has previously been shown,^5^, ^11^ APP associates with mitochondrial translocation channels in transit. We used immunoprecipitation with antibodies against Tom40, a core component of the protein import channel of the mitochondrial outer membrane and observed that under GMP‐1 treatment conditions amount, coimmunoprecipitated APP decreased by 50% in comparison with DMSO treatment (Figure 2A and B). We have studied the effect of GMP‐1 treatment on distribution of APP in isolated mitochondria and light membrane fractions (P3). The results in Figure 2C and D show that amount of mitochondria‐associated APP is decreased by 50% in GMP‐1‐treated cells as compared to DMSO accompanied by increase in mature APP in P3 fraction. We have previously shown that mitochondrial APP staining with antibodies against N‐terminal epitope of APP molecule resulted in detection of several APP fragments of apparent molecular weight of 20‐75 kD.^11^ Results presented in Figure 2 suggest partial inhibition of APP association with mitochondria upon GMP‐1 treatment and APP redistribution between different intracellular compartments.

### 3.3 Effect of GMP‐1 treatment on viability and motor function of transgenic drosophila expressing Aβ_1‐42_ as well as tandem Aβ_1‐42_ (T‐Aβ_1‐42_)

**Figure 3:**
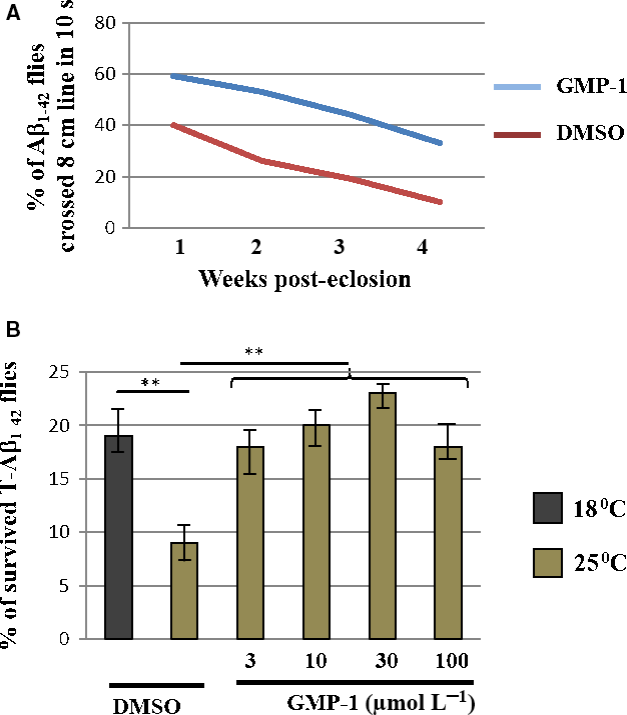
Effect of GMP‐1 treatment in drosophila expressing Aβ_1‐42_ variants in CNS. A, Mobility climbing assay in Aβ_1‐42_‐expressing flies in the presence of DMSO or 50 μmol/L of GMP‐1. Six independent measurements were performed. B, Viability assay of T‐Aβ_1‐42_ flies growing at permissive 18°C and non‐permissive 25°C temperatures in the tubes with added DMSO or increasing concentrations of GMP‐1. Bars represent percentage of T‐Aβ_1‐42_ homozygotes F1 offsprings to the total number of hatched flies. Quadruplicate measurements were performed **P < 0.01, n=4

Drosophila flies are widely used in initial drug screening programmes as simple, fast and inexpensive in vivo model to assess drug toxicity and efficacy. Because of the lack of blood‐brain barrier, drosophila models allow to study CNS effect of drugs ingested from the food. We have used two previously described fly models expressing Aβ_1‐42_ as well as more aggregation‐prone and toxic tandem Aβ_1‐42_ (T‐Aβ_1‐42_) in CNS. Transgenic drosophila expressing Aβ_1‐42_ in CNS had reduced mobility in comparison with wild‐type flies.^24^ Figure 3A shows that the treatment of Aβ_1‐42_‐expressing flies with 50 μmol/L of GMP‐1 rescued motility defects associated with brain Aβ_1‐42_ expression. We have also used a T‐Aβ_1‐42_ drosophila model to assess the effect GMP‐1 on toxicity of aggregation‐prone Aβ species. It has been previously shown that hatching of T‐Aβ_1‐42_‐expressing flies was compromised at 25°C in comparison with 18°C incubation temperatures.^25^ We have observed that addition of GMP‐1 into the food of T‐Aβ_1‐42_‐expressing flies growing at 25°C rescued hatching to the levels similar to 18°C (Figure 3B). These results suggest neuroprotective effect of GMP‐1 in drosophila Aβ_1‐42_‐expressing models.

### 3.4 GMP‐1 treatment of 5xFAD transgenic mice. Effect on behaviour, memory, amyloid beta accumulation, neuroinflammation and mitochondrial function

**Figure 4:**
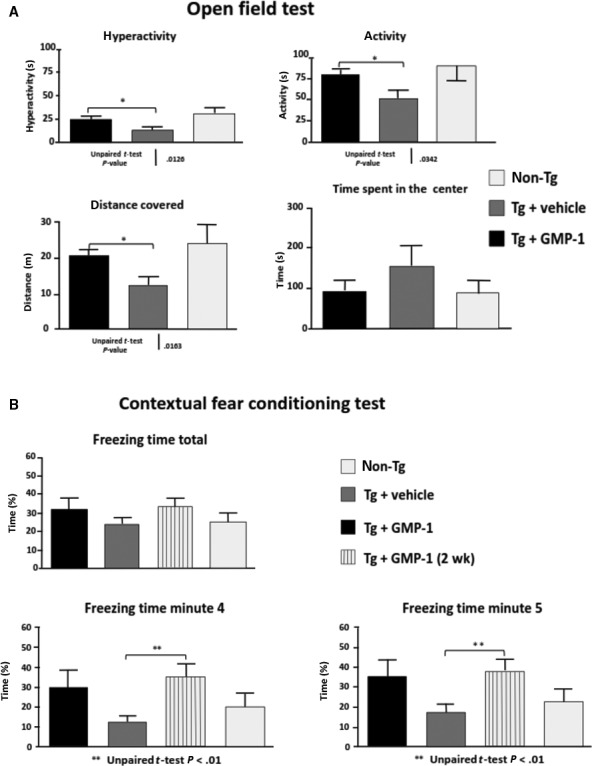
Effect of GMP‐1 treatment on behaviour and memory in 5xFAD transgenic mice model. A, Results of open field test (means ± SEM) in different mice groups. *P < .05, n = 15. B, Results of contextual fear conditioning test (means ± SEM) in four different mice groups. **P < .01, n = 15

**Figure 5:**
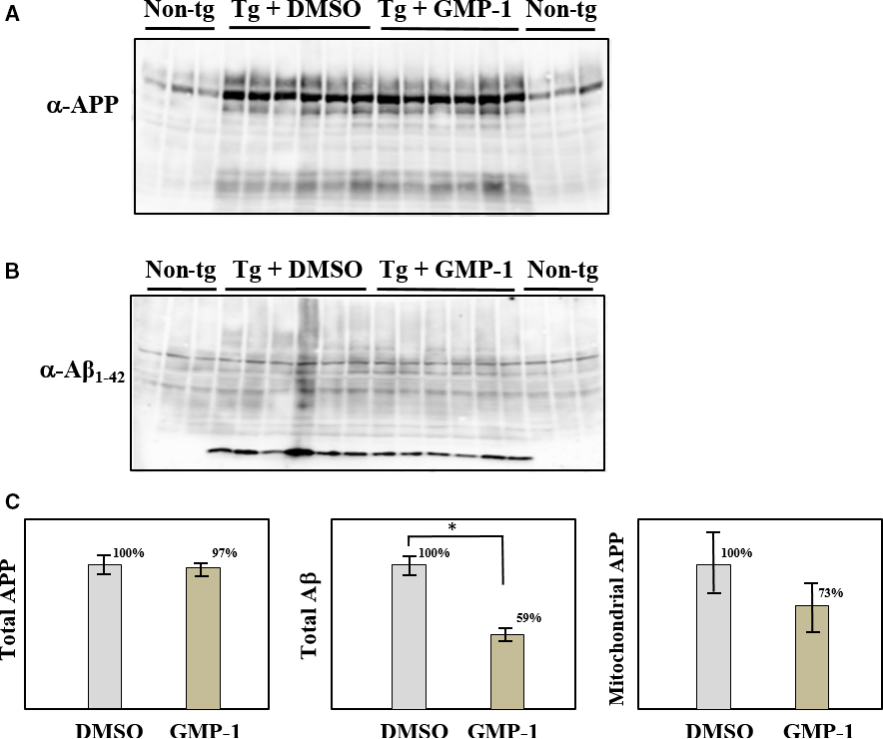
Effect of GMP‐1 treatment on total APP and Aβ as well as mitochondria‐associated APP. A, Western blot of total brain extract using anti‐APP (22c11) antibodies. B, Western blot of total brain extract using anti‐Aβ_1‐42_ antibodies. C, Quantification results of Western blot staining. Results (means ± SEM) are represented as percentages of signals measured in placebo‐treated mice vs GMP‐1‐treated mice. *P < .05, n = 6

**Figure 6:**
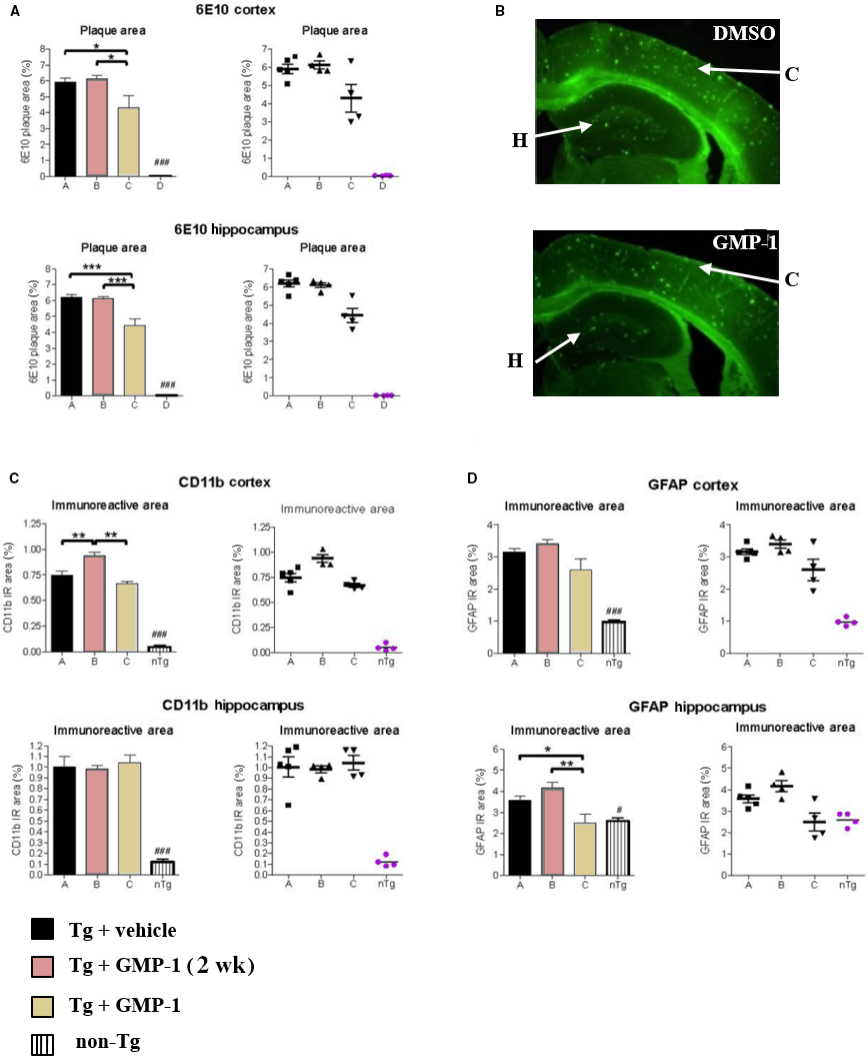
Immunohistochemical assessment of GMP‐1 treatment on amyloid plaques accumulation and neuroinflammation. A, Amyloid plaque area in cortex and hippocampus measured as anti‐APP (6E10) antibody staining. Placebo‐treated, GMP‐1 chronically treated, GMP‐1 acutely treated 5xFAD mice as well as non‐transgenic animals indicated as A, B, C and D, respectively. Differences between groups were calculated by one‐way ANOVA followed by a Newman‐Keuls post‐hoc test, the alpha‐error set to 0.05. *P < .05, ***P < .001, n = 6. B, Representative images of brain slices from 6‐month‐old 5xFAD mice stained with 6E10 antibodies and Alexa Fluor^®^ 488 secondary antibodies. Arrows indicate area of cortex (C) and hippocampus (H). C, Astrocytosis in the cortex and hippocampus measured as anti‐GFAP antibody staining. Placebo‐treated, GMP‐1 chronically treated, GMP‐1 acutely treated 5xFAD mice as well as non‐transgenic animals indicated as A, B, C and D, respectively. Differences between groups were calculated by one‐way ANOVA followed by a Newman‐Keuls post‐hoc test, the alpha‐error set to 0.05. *P < .05, **P < .01, n = 6. D. Microglia activation in the cortex and hippocampus measured as anti‐CD11b antibody staining. Placebo‐treated, GMP‐1 chronically treated, GMP‐1 acutely treated 5xFAD mice as well as non‐transgenic animals indicated as A, B, C and D, respectively. Differences between groups were calculated by one‐way ANOVA followed by a Newman‐Keuls post‐hoc test, the alpha‐error set to 0.05. **P < .01, n = 6

**Figure 7:**
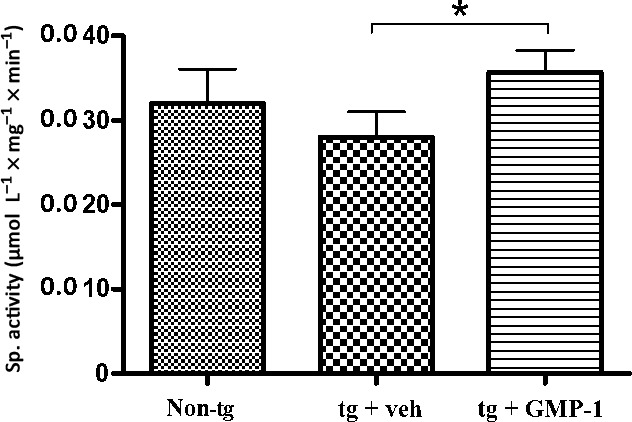
Cytochrome oxidase activity measurement in isolated brain mitochondria. Result bars represent specific activity (means ± SEM) of cytochrome oxidase measured as described in Materials and Methods. *P < .05, n = 6

The 5xFAD model is a double transgenic APP/PS1 mouse model that coexpresses five AD mutations leading to accelerated plaque formation and increased Aβ_1‐42_ production.^26^ Amyloid deposition and gliosis begin at 2 months accompanied by robust neuronal loss starting from 3 months of age as well as cognitive and memory decline.^26^ Moreover, Devi and Ohno have reported mitochondrial dysfunction in the brains of 5xFAD mice and found full‐length APP as well APP C‐terminal fragment C99 in isolated brain mitochondria fractions.^27^ Four groups of 15 animals were used as follows: tg mice having 0.5% DMSO in their drinking water ad libitum as placebo; 5xFAD tg mice that received 16.7 mg/kg of compound in the drinking water from 3 weeks of age until the end of experiment at 6 months of age; tg mice that received 16.7 mg/kg of compound in the drinking water at the age of 5.5 months during 2 weeks; non‐tg littermates receiving 0.5% DMSO in drinking water. Because of its physicochemical properties such as molecular mass of 229 Da, XLogP coefficient 1.67, small polar surface area GMP‐1 was predicted to penetrate blood‐brain barrier by several ADME predictors. Therefore, oral administration route was chosen and the dose corresponded to the 100 μmol/L of GMP‐1 dissolved in water. Both acute and chronic treatments with GMP‐1 were safe—no differences between different treatment groups were observed in Irwine general health test, body mass or internal organs evaluation (data not shown). Results of behaviour and memory tests are presented in Figure 4. Results of the open field locomotion test show that 5xFAD mice are less active and spend more time in the centre of the box in comparison with their wild‐type littermates indicating their increased anxiety behaviour. GMP‐1‐treated 5xFAD mice exhibited restoration of locomotion and explorative behaviour to the levels of wild‐type littermates (Figure 4A). Next, we tested 5xFAD mice with hippocampus‐dependent contextual fear conditioning, in which mice learn to associate a distinct context with an aversive foot shock. Wild‐type mice exhibited a robust conditioned fear response as assessed by freezing (the absence of all but respiratory movements) when placed back into the conditioning chamber 1 day after training. Both acute and chronically GMP‐1‐treated 5xFAD mice exhibited higher levels of freezing as compared to untreated animals especially when calculating freezing time at minutes 4 and 5 (Figure 4B). These results indicated restorative effect of GMP‐1 treatment on hippocampus‐dependent memory formation. To understand underlying mechanism of GMP‐1 effect on mice memory and behaviour, we have performed analysis of brain APP and Aβ content as well as immunohistochemical analysis of inflammatory markers and mitochondrial function. Analysis of total APP in the brain homogenate showed no differences between GMP‐1‐ and placebo‐treated groups, whereas Aβ_1‐42_ levels were significantly lower in the GMP‐1‐treated group (Figure 5). The levels of APP associated with brain‐isolated mitochondria, albeit lower in the GMP‐1‐treated group, did not reach statistical significance (Figure 5C). In two brain regions, cortex and hippocampus, we immunohistochemically assessed amyloid beta plaque load as well as astrocytosis and microglia activation. Results presented in Figure 6A indicate that the Aβ plaque load in the cortex and hippocampus decreased in the 5xFAD group chronically treated with GMP‐1 in comparison with placebo‐treated and acutely treated GMP‐1 group. Figure 6B shows representative images of brain sections containing cortex and hippocampus stained with 6E10 antibodies. In brain tissue of 6‐month‐old 5xFAD mice, 6E10 antibodies recognized predominantly plaque pathology despite the fact that they can react with full‐length APP. GMP‐1 treatment significantly reduced number of plaques and antibody‐stained area both in the cortex and hippocampus as compared with placebo‐treated animals. The level of astrocytosis measured as GFAP staining was significantly decreased in hippocampal sections of 5xFAD group chronically treated with GMP‐1 in comparison with placebo‐treated and acutely treated GMP‐1 group (Figure 6C). Microglia activation levels measured as CD11b staining were significantly reduced in the cortex of acutely and chronically GMP‐1‐treated 5xFAD groups as compared to placebo‐treated 5xFAD mice (Figure 6D). Finally, we assessed brain mitochondrial function by measurement of cytochrome *c* oxidase activity of isolated brain mitochondria. We found that cytochrome *c* oxidase activity is significantly increased in GMP‐1‐treated 5xFAD mice in comparison with placebo‐treated group (Figure 7).

## 4 DISCUSSION

Several studies link mitochondrial dysfunction in neurodegenerative disorders with abnormal accumulation of disease‐associated proteins in mitochondria.^2^, ^3^, ^4^, ^5^, ^6^ In this view, targeting intracellular protein trafficking and preventing abnormal accumulation of disease‐related proteins in mitochondria hold a promise as a new therapeutic strategy for various neurodegenerative disorders.^28^ Molecular chaperones Hsp70 and Hsp90 regulate variety of cellular processes including general protein folding, degradation and trafficking.^29^ They have been described as potential targets for the treatment of neurodegenerative disorders; however, direct activation or inhibition of Hsp70/Hsp90 could lead to undesirable side‐effects especially in lifelong treatment scenario. The action of molecular chaperones Hsp70/Hsp90 is mediated by the co‐chaperones, proteins interacting with molecular chaperones and providing their functional specificity.^30^ Disruption of specific protein‐protein interactions within molecular chaperone network would not elicit major disturbance in molecular chaperone system. Here, we described development of inhibitors of molecular interaction between Hsp70/Hsp90 and Tom70 a mitochondrial protein import receptor and TPR domain co‐chaperone. We have used in silico docking to identify molecules that have capacity to interact with TPR domain of Tom70 and inhibit its interaction with the Hsp70/Hsp90. The identified compound, 2‐(methoxymethyl)pyrimido[1,2‐a]benzimidazol‐4‐ol, GMP‐1, was found to compete with Hsp90‐derived peptide for Tom70 binding. It has to be mentioned that GMP‐1 interaction with TPR domains has relatively low affinity for Tom70 as well as relatively low specificity towards other TPR proteins such as PP5, FKBP51 and Tom34. We are currently executing hit‐to‐lead programme to identify compounds with improved affinity/specificity as well as their drug‐likeness. GMP‐1 analogues have previously been patented and described as CNS drugs that have depressing and ataractic effect and could be used to relieve anxiety and tension states (patent US4109087 A, 1978). They also reduced local inflammation measured by carageenan oedema assay and delayed hypersensitivity test in rats (patent US4109087 A, 1978). It is well known that progressive Aβ accumulation in the brain triggers neuronal hyperexcitation both in AD animal models and patients with AD often resulting in seizures^31^, ^32^; therefore, compounds inhibiting such overexcitation could have neuroprotective effect. Our results in 5xFAD mice open field test indicate that GMP‐1 could relieve anxiety state to the levels seen in wild‐type animals. Also GMP‐1‐treated animals had reduced levels of astrocytosis and microglia activation in comparison with placebo‐treated mice indicating reduced neuroinflammation. It is not currently known whether the reduced brain inflammation is linked to reduced brain Aβ load or it exerts an independent effect on astrocytes and microglial cells. We have unexpectedly observed reduced levels of total Aβ and amyloid plaques upon GMP‐1 treatment. One plausible explanation would suggest that modulation of molecular chaperone network can also modify molecular chaperone involvement into intracellular Aβ degradation. With regard to mitochondrial metabolism, we have observed restoration of cytochrome oxidase activity in the GMP‐1‐treated mice. We have also found reduced levels of mitochondria‐associated APP in the GMP‐1‐treated mice; however, because of the relatively low number of studied animals, the values did not reach statistical significance. Finally, we have proposed a new treatment strategy for AD protecting mitochondrial function via modulation of molecular chaperone network. GMP‐1 exhibits neuroprotective effect in drosophila and mice AD models resulting in increased fly viability and memory improvement in 5xFAD mice.

## CONFLICT OF INTERESTS

Authors report no conflict of interests.

## AUTHORS’ CONTRIBUTION

P.P. contributes with planning and performing experiments, analysing data and manuscript preparation. B.H.P. contributes with planning and performing experiments, M.W. contributes with experimental planning, B.W. contributes with experimental planning, manuscript preparation.

## ADDITIONAL INFORMATION

The patent P60490GB has been obtained for GMP‐1. GreatMatterPharma AB is a patent holder.
